# A Geometric Representation of Collective Attention Flows

**DOI:** 10.1371/journal.pone.0136243

**Published:** 2015-09-01

**Authors:** Peiteng Shi, Xiaohan Huang, Jun Wang, Jiang Zhang, Su Deng, Yahui Wu

**Affiliations:** 1 Science and Technology on Information Systems Engineering Laboratory, National University of Defense Technology, Changsha, China; 2 School of Systems Science, Beijing Normal University, Beijing, China; Beihang University, CHINA

## Abstract

With the fast development of Internet and WWW, “information overload” has become an overwhelming problem, and collective attention of users will play a more important role nowadays. As a result, knowing how collective attention distributes and flows among different websites is the first step to understand the underlying dynamics of attention on WWW. In this paper, we propose a method to embed a large number of web sites into a high dimensional Euclidean space according to the novel concept of flow distance, which both considers connection topology between sites and collective click behaviors of users. With this geometric representation, we visualize the attention flow in the data set of Indiana university clickstream over one day. It turns out that all the websites can be embedded into a 20 dimensional ball, in which, close sites are always visited by users sequentially. The distributions of websites, attention flows, and dissipations can be divided into three spherical crowns (core, interim, and periphery). 20% popular sites (Google.com, Myspace.com, Facebook.com, etc.) attracting 75% attention flows with only 55% dissipations (log off users) locate in the central layer with the radius 4.1. While 60% sites attracting only about 22% traffics with almost 38% dissipations locate in the middle area with radius between 4.1 and 6.3. Other 20% sites are far from the central area. All the cumulative distributions of variables can be well fitted by “S”-shaped curves. And the patterns are stable across different periods. Thus, the overall distribution and the dynamics of collective attention on websites can be well exhibited by this geometric representation.

## Introduction

In each second, 684478 pieces of content will be shared on Facebook, 204166667 emails will be sent, 100000 tweets will be posted, 27778 new posts will be published on Tumblr, and 571 new websites will be created, data keeps growing with no signs of stopping [1]. However, only 3 billion users (2014) consume this ever-accumulating information on the Internet [2], we are drowning in the sea of information and data. As pointed out by H.A. Simon, “a wealth of information creates a poverty of attention” [3], attention will doubtlessly play more and more important roles in the near future because of its scarcity and the overload of information. Thus, how attention efficiently allocates among the over-abundant information resources is of overwhelming importance [3].

Collective attention as a fast booming area has attracted much attention of researchers since the seminal works of B.A. Huberman et al [4–7]. Previous studies focus on the micro-level dynamics of collective attention among many information sources, including decaying [8], competing [9], and switching [10]. Besides popularity of information resources, the connections between contents are also important for the dynamic allocation of continuous attention flows. Clickstream [11] defined as an ordered sequence of web page or resource viewed by a user can be treated as an embodiment of a continuous attention flow [12]. In fact, many scholars have highlighted the huge application potentials behind clickstream data, such as categorizing visitors into different groups based on visitors trajectory [13], predicting customers’ choice behaviors by using clickstream data in one shopping website [14, 15], developing interactive visualization tools to present interactions between users and websites [16, 17].

However, most of traditional clickstream studies only focus on single website, especially in E-commercial field. The allocation of attention flows on the World Wide Web scale is seldom addressed in previous studies. The first reason accounting for the lack of this study is the techniques and methods for analysing large scale flow data are in short because most of conventional network analysis methods only care about connection topology but not the flow information on it. For example, the landmark conclusions on the heterogeneity [18] and the diameter of WWW [19] is made by the hyper-link connection structure of websites. Nevertheless, as the fast development of techniques like search engine, bookmarks, default pages, a large number of user transitions are not along hyper-links [20]. Therefore, the behavioral data on the entire WWW level is needed to know how websites connect. But the problem is collecting clickstream data on the entire WWW level is still very difficult.

In this paper, we try to give an overall picture of how collective attention distributes among websites and how websites are connected by clickstreams. The raw data collected by Indiana University is on the net gate level of the whole university. All clickstreams are generated by faculties and students within the campus. Although the data is apparently biased, it covers considerable range of WWW: the average number of unique websites per day appeared in our data is 123137, and the average total amount of daily traffic is 45563567. This high quality and detailed data enables us to study attention allocating and flowing between sites on a very large scale.

Second, we use the so called open flow network [21] to model clickstreams. An open flow network is a special directed weighted network in which nodes are websites and weighted links are directed traffics between two sites [18]. Two special nodes, the source and the sink are added to model the environment, i.e., the off-line world. Therefore, the open flow network cannot only represent the topology of WWW and the collective behaviors of users, but also consider the flow exchanged between the system and the off-line world, i.e., the inward (flow from the source) and outward flows (dissipation, the flows to the sink). These flows cannot be neglected because a large fraction (almost 56%) of attention flows is dissipation (go off-line) for all nodes [22]. Further, a bonus property of adopting open flow network model is the flow balancing, i.e., the inflow equals the total out flow for all nodes if the flows are supposed to be in steady state.

Flow distance is a novel conception that developed for open flow networks which combines the consideration of network topology and collective behavior of users. It is defined as the average number of steps of users jumping between two nodes along all possible (directed and indirected) paths. With this notion, we can embed the whole flow network into a Euclidean space such that the Euclidean distance equals to the flow distance between any two nodes pair. In this geometric representation, we can clearly see that the distribution of websites and attention flows forms a ball where each site locates in a unique position surrounded by tightly connected websites. We further quantitatively investigate the distribution patterns of sites, attention flows and dissipations, and find that the cumulative quantities can be well fitted by “S”-shaped curves. Accordingly, all websites can be grouped into three layers. Popular websites like Google.com, Myspace.com, etc. are in the core where a large fraction of attention flows and a relative small fraction of dissipations are attracted. In the interim, a large number of websites with a small proportion of attention flows and a relative large fraction of dissipations locate. And other small sites with a few traffics and dissipations locate in the periphery. All the observations are stable along time.

## Methods and Materials

### Data

The raw data that we employed is from the clickstream data of Indiana university campus(http://cnets.indiana.edu/groups/nan/webtraffic/click-dataset/), which records the surfing behaviors of the users in Indiana University during the period 2006.10-2008.02 [20, 23]. Although this data set is a biased sample of the entire WWW [20, 24–27], it contains 123137 websites and 45563567 traffics one day in average, especially the top websites during 2006.10-2008.02 are included, like Myspace.com, Facebook.com, Yahoo.com, and so on. Therefore, a clear overall picture can be obtained, and the results are representative.

We adopt the classification of websites in (http://sitereview.bluecoat.com/sitereview.jsp). There are a lot of advertising websites in the raw data, like 2o7.net, Advertising.com, Doubleclick.com, and so on. We identify them according to the classification data and remove them from the raw data.

### Construction of open flow networks

In the raw data, the switches between two websites are recorded. The basic format of one record is like this: (time stamp, referrer, host, path), where the time stamp is the unix time of the surfing behavior. Referrer and host are domain names, and path is the visiting location of the website.

We construct an open flow network model [28, 29] for all the records in one day. First, we parse all the records in the data set, and extract their domain names. We maintain a dictionary for storing all the distinct domain names (websites), and replace the domain name strings in the raw data (referrer and host) by the index of the website in the dictionary. Second, we ignore time stamps and count the total number of transitions *f*_*ij*_ for the given pair of web site indices, *i* and *j*. This is the flow from *i* to *j*. Further, there are some null strings in the referrer records in the raw data representing that these transitions have no or missing referrers. We treat these records as the flows from the source (the outside world), thus the null string represents the source. Third, we balance the entire network by adding dissipation flows from all nodes to the sink such that all the inflow balances with the out flow for each node. The dissipations must be added manually because all the jumps to off-line world are not recorded in the raw data.

Finally, we can obtain an (*N* + 2) × (*N* + 2) flux matrix denoted as *F*, where *N* is the total number of websites,

$$\begin{array}{c} F_{\left(N+2)\times (N+2\right)}={\left\{f_{ij}\right\}}_{\left(N+2)\times (N+2\right)}, \end{array}$$ (1)

In which, node 0 represents the source, and node *N* + 1 represents the sink. Therefore, the flow *f*_*i*, *N* + 1_ is the dissipation of site *i*, and $\sum_{i=1}^{N+1}f_{ij}$ is the total attention flow (traffic) of *i*. We also call this flux matrix as attention flow network.

The unique advantage of the open flow network and the distinction from conventional topological network and closed flow network are the consideration of flows between site s, especially the flows from the source and to the sink.

### Flow distances

Many scholars have paid attention to distances on a network, like the shortest path distance [30], and the mean first-passage distance derived by the random walk model [31–34]. While due to the existence of the source and the sink, the conventional methods for computing the random-walk distance could not be directly applied to the open flow network. Therefore, we must develop a new method to calculate the flow distance. First, we will calculate a markov matrix according to the original flow matrix,

$$\begin{array}{c} m_{ij}=\frac{f_{ij}}{\sum_{k=1}^{N+1}f_{ik}}, \end{array}$$ (2)

Where, *M* is the markov transition matrix, and *m*_*ij*_ represents the probability of a user jumping from *i* to *j*.

The flow distance between two websites is defined as the average distance that one visitor jumping from *i* to *j* for the first time along all possible flow pathways [21]. The closer the websites, the easier is for visitors’ jumping from one website to another. According to [21], the flow distance between two websites is *l*_*ij*_:

$$\begin{array}{c} l_{ij}=\frac{{\left(MU^{2}\right)}_{ij}}{{\left(U\right)}_{ij}}-\frac{{\left(MU^{2}\right)}_{jj}}{{\left(U\right)}_{jj}}, \end{array}$$ (3)

Where, *U* = *I* + *M* + *M* ^2^ + ⋯ = (*I*−*M*)^−1^, (*U*)_*ij*_ is the pseudo-probability from *i* to *j* along all possible paths and *I* is the unit matrix with *N* + 2 nodes.

**Fig 1:**
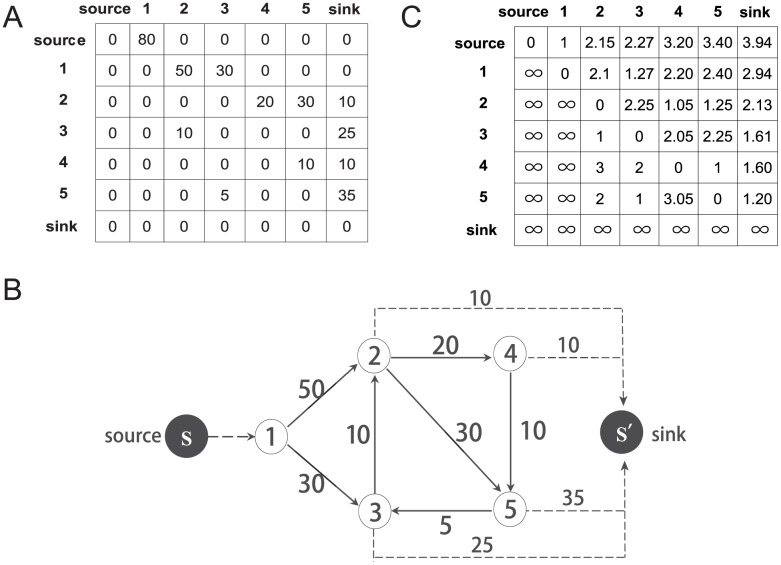
An example attention flow network including 7 nodes. (A)The flux matrix of the example network. Noting that the summation of all the elements in the *ith* column equals to the summation for the *ith* row except “source” and “sink”, thus, the network is balanced. (B) The original example open flow network. (C) shows the flow distances among websites, where infinity means that there is no connected path from *i* to *j*.

Next, we will apply our method on an example open flow network to interpret the calculation of flow distances, as shown in Fig 1.

In Table 1, we compare three kinds of distances for the example network. First, the shortest distances based on the binary link structure always under estimate the walk distances for real users because the shortest distances assume that all users can find the shortest path in the entire network level. Second, we compare the random walk distances defined as the average steps along all possible flow paths that a random walker jumping along the links on the closed version of the same flow network. In the closed flow network, the source and the sink are excluded, and the jumping probability from *i* to *j* is just the ratio between the flux *f*_*ij*_ and the total out flow ($\sum_{j=1}^{N+1}f_{ij}$). Thus, the dissipation of all nodes are not considered. In this way, the random walk distances of the closed flow network always over estimate the average path lengths. However, real users always not travel too long paths because they are very likely to get offline in each jump. Thus, flow distance can depict the average distance among websites more accurately by considering almost all information of the network [21].

**Table 1:** The comparisons between flow distances and other well-known distances.

	**1 → 3**	**2 → 3**	**1 → 4**	**2 → 4**
Shortest Distance	1	2	2	1
Random Walk Distance	2.5	2.4	6.875	5.5
Flow Distance	1.274	2.25	2.2	1.055

However, the flow distances matrix *L* is not symmetric, but the embedding into the Euclidean space requires symmetric distances. Thus we calculate symmetric flow distances *C* as:

$$\begin{array}{c} c_{ij}=l_{ij}+l_{ji}, \end{array}$$ (4)

This is a measure for averaging *l*_*ij*_ and *l*_*ji*_. And it can be also explained as the average commuting distance [22] which is the average path length for a random walker going from *i* to *j* and finally returning back to *i* again.

### The embedding of websites

According to the symmetric flow distances (*c*_*ij*_), we can embed websites into a Euclidean space by imposing the Euclidean distances equalling the flow distances among websites as accurately as possible. Each node in the Euclidean space has a “geometric image” of the websites. We adopt the reduced version of the Bigbang algorithm [35] to embed.

The algorithm implementation is shown as the following steps.

**Step1: Initialization phase: assign a random position $v_{k}^{d}$ denoting the coordinate vectors of website *k* in d-dimensional space for each website in d-dimensional space.**

**Step2: Adjustment phase: compute each node’s position according to the spring algorithm [36] such that the embedding errors for all pairs of websites $E_{ij}^{\left(1\right)}(V_{i},V_{j})$ are small enough.**

In which,

$$\begin{array}{c} E_{ij}^{\left(1\right)}\left(V_{i},V_{j}\right)=∥V_{i}^{d}-V_{j}^{d}∥-c_{ij} \end{array}$$ (5),

where $∥V_{i}^{d}-V_{j}^{d}∥$ is the norm of the vector $V_{i}^{d}-V_{j}^{d}$ denoting the Euclidean distance between websites pairs(*i*, *j*), and *c*_*ij*_ is the symmetric flow distance between *i* and *j*. $E_{ij}^{\left(1\right)}(V_{i},V_{j})$ denotes the difference between the Euclidean distance and the flow distance.

We will use the spring algorithm to compute the positions. Suppose that any two websites are connected by a spring and the relaxed length of each spring equals to the flow distance *c*_*ij*_, if the distance of two websites in the d-dimensional space is larger than the relaxed length of the spring, the websites will exert a pulling force. Otherwise, there will be a repulsive force between them. The size of the force is proportional to value of $E_{ij}^{\left(1\right)}(V_{i},V_{j})$. This step will repeat until the total embedding error(denoted by $\bar{d}$, the average embedding distortion, which is introduced in the **Step3**) is reduced to a given threshold level (in this paper, the level is set to 1.50).

**Step3: Fine-tuning phase: fine-tune the positions according to the embedding distortions, denoted by $E_{ij}^{\left(2\right)}$:**

$$\begin{array}{c} E_{ij}^{\left(2\right)}={\left(d_{ij}-1\right)}^{2}, \end{array}$$ (6)

where

$$\begin{array}{c} d_{ij}=\max\left(\frac{∥V_{i}-V_{j}∥}{c_{ij}},\frac{c_{ij}}{∥V_{i}-V_{j}∥}\right) \end{array}$$ (7)

depicts the max ratio of the Euclidean distance and the flow distance. The effectiveness of the embedding method can be characterized by $\bar{d}$:

$$\begin{array}{c} \bar{d}=\left(\sum_{i=1}^{N}\sum_{i=1}^{N}d_{ij}\right)/N^{2}, \end{array}$$ (8)

Repeat adjusting the positions of nodes by using the spring algorithm, until $\bar{d}$ (the average embedding distortion between websites) is smaller than 1.14.

Actually, the first adjustment phase makes large adjustments quickly while the second phase modulates the websites positions slightly when the value difference between Euclidean distance and flow distance is small. Taking the ratio of Euclidean distance and flow distance as an important indicator is to eliminate the effect of the value size of distances.

## Results

### The distribution of flow distances among websites

**Fig 2:**
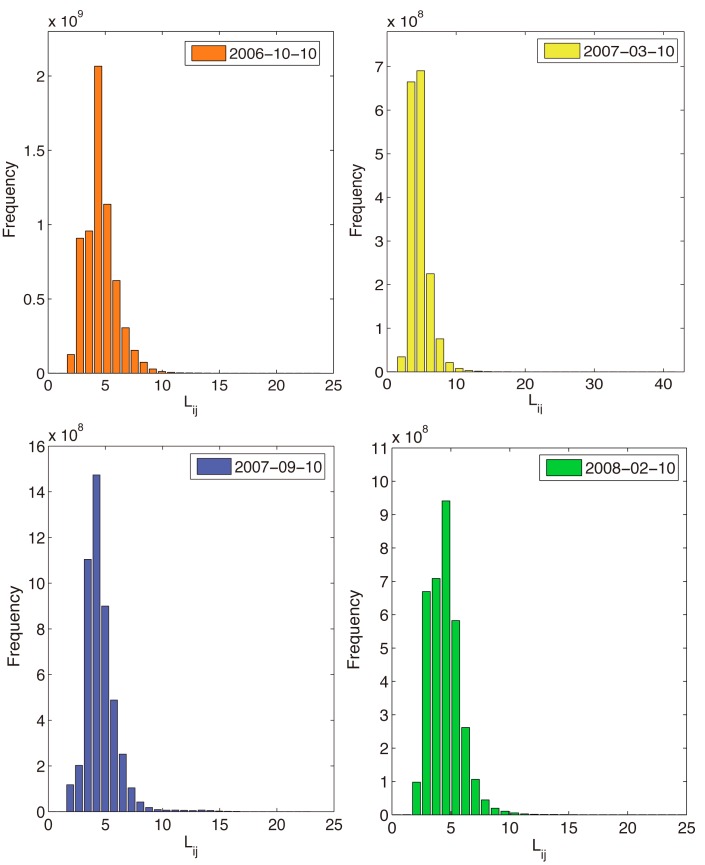
The distribution of flow distances (*l*_*ij*_) on October 10, 2006, March 10, 2007, September 10, 2007, and February 10, 2008.

We use the open flow network to model the clickstream data of October 10,2006 (158232 nodes included), March 10,2007 (85080 nodes included), September 10,2007 (138047 nodes included), and February 10,2008 (111189 nodes included) (see Method section) and we calculate the flow distances *l*_*ij*_s (see Method section) for all node pairs of the flow network. The distributions of all flow distances are shown in Fig 2. We find that they are similar in different times and the average distances in four snapshots are all close to 4.5 which exhibiting the small world effect. The flow distance notion both considers the topological closeness of websites and the average real behaviors of surfing which is apparently different from the traditional shortest path distance [30] and random walk distance [31–34] on close flow networks (see the detailed discussions in the method section).

### The geometric representation of websites

**Fig 3:**
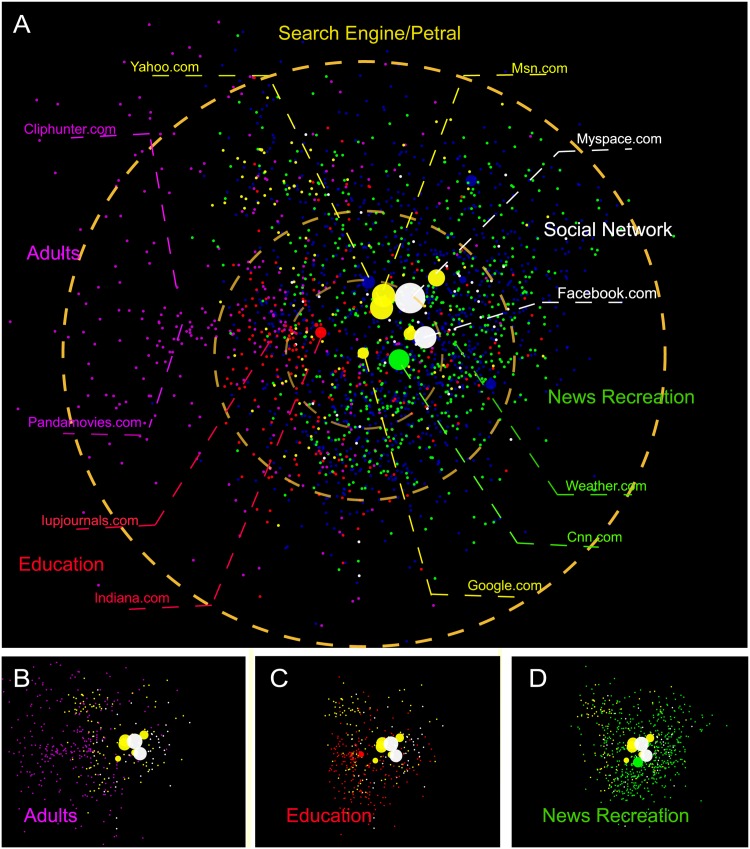
The geometric representation of top 2200 websites. Sub figure A shows the **geometric representation** of the selected websites on October 10 in 2006. The node colors represent the categories of the websites and the node sizes are proportional to the traffics of the focus websites. The small figures in the bottom are the same representations for selected Adults websites in B, Education websites in C, and News Recreation websites in D.

Next, we select a strong connected sub-network containing 2200 websites from the top 4000 websites of the original network on October 10,2006 to be embeded into a 20 dimensional Euclidean space by using a reduced version of BigBang algorithm [35] (details can be referred to the method section) such that the Euclidean distance between any two nodes is as closed as possible to their flow distance. In this way, each node obtains a coordinate. To visualize this sub-network, we project all nodes into a two dimensional space by using the PCA method [37, 38] to reduce the dimensionality as shown in Fig 3.

**Fig 4:**
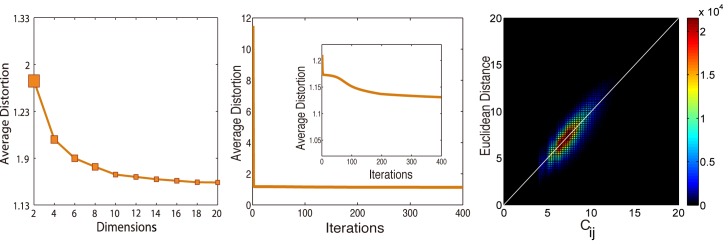
The effectiveness of the embedding. (A) The average distortion of embedding algorithm decreases with the embedding dimension. (B) The average distortion of embedding algorithm in different iterations. The inset shows the enlarged lower left corner of the line. (C) shows the relationship between all the Euclidean distances and the flow distances as the final best results of the embedding.

By using websites category data provided by Blue Coat Systems, Inc., we classify websites into 6 classes according to their domain names. And the sites with same class may show similar contents which are always visited sequentially by users. This phenomenon can be observed in Fig 3D for classes of News ecreation, Fig 3C Education and Fig 3B Adults because they locate three distinct regions of the map. However, other sites like search engines, social networks always provide synthetic contents or services, such that they scatter in the space all around. Fig 4 shows the embedding effectiveness analysis. A demonstrates that the average distortion decreases with the embedding dimensions. B shows the variation of average distortion during the iterations. C gives the comparison between the Euclidian distance and *C*_*ij*_.

The positions of websites can reveal their centrality in the whole network. The sites locating in the central areas are always more important than other sites for the whole websites ecosystem because the distances from the central sites to others being small implies that users may visit the central websites frequently wherever they come from or go to. It is interesting that although the social network sites like Myspace.com, Facebook.com attract very large amount of traffics, they are not the center of the whole system. Instead, Google.com is more central in the sense of attention flow positions than the social networks (see Table 2). This observation is consistent with our intuition that Google.com has become the portal of the whole web world and to transport users’ attention into the virtual world.

**Table 2:** Top 15 websites with shortest average distance.

**Rank**	**Websites Name**	**Average Distance**	**Total Traffic**
1	*Google*.*com*	4.2623	(9)513580
2	*Yahoo*.*com*	4.8258	(4)1052668
3	*Myspace*.*com*	5.1442	(1)2769875
4	*Indiana*.*edu*	5.2725	(11)510071
5	*Aol*.*com*	5.3595	(8)582965
6	*Msn*.*com*	5.4158	(3)1059774
7	*Ebay*.*com*	5.4925	(7)612550
8	*Iupui*.*com*	5.5652	(20)152432
9	*Wikipedia*.*org*	5.5681	(27)66868
10	*Forbes*.*com*	5.6306	(105)12559
11	*Comcast*.*net*	5.6341	(74)29550
12	*Ask*.*com*	5.6350	(53)19372
13	*Cnn*.*com*	5.6531	(5)946587
14	*About*.*com*	5.6670	(64)22094
15	*Dogpile*.*com*	5.6670	(272)4282

The numbers in the parentheses are the ranking orders according to the focus indicators.

Table 2 lists top 15 websites ranked by the average distance to other nodes in a decreasing order. As a comparison, we also listed the ranking results by PageRank algorithm [39], which are shown in Table 3. We find that PageRank tends to give high ranks to the websites with more in-links (Indiana.edu, Amazon.com, etc.), but not high attention flows (Myspace.com, Msn.com, Cnn.com, Aol.com, and so on).

**Table 3:** Top 15 websites with highest PageRank.

**Rank**	**Websites Name**	**PageRank**	**Total Traffic**
1	*Indiana*.*edu*	0.0554	(11)510071
2	*Yahoo*.*com*	0.0323	(4)1052668
3	*Yimg*.*com*	0.0283	(6)777032
4	*Amazon*.*com*	0.0184	(13)330735
5	*Iupui*.*edu*	0.0139	(20)152432
6	*Imdb*.*com*	0.0137	(29)58286
7	*Indystar*.*com*	0.0132	(32)55404
8	*Photobucket*.*com*	0.0128	(15)261927
9	*Google*.*com*	0.0125	(9)513580
10	*Yourfreedvds*.*com*	0.0123	(235)4979
11	*Gannettonline*.*com*	0.0115	(130)10570
12	*Mate* 1.*com*	0.0092	(164)8020
13	*Monster*.*com*	0.0078	(75)19332
14	*Aol*.*com*	0.0076	(8)582965
15	*Go*.*com*	0.0072	(14)268620

The numbers in the parentheses are the ranking orders according to the focus indicators.

### The distributions of attention flows, attention dissipations and websites in the geometric representation

**Fig 5:**
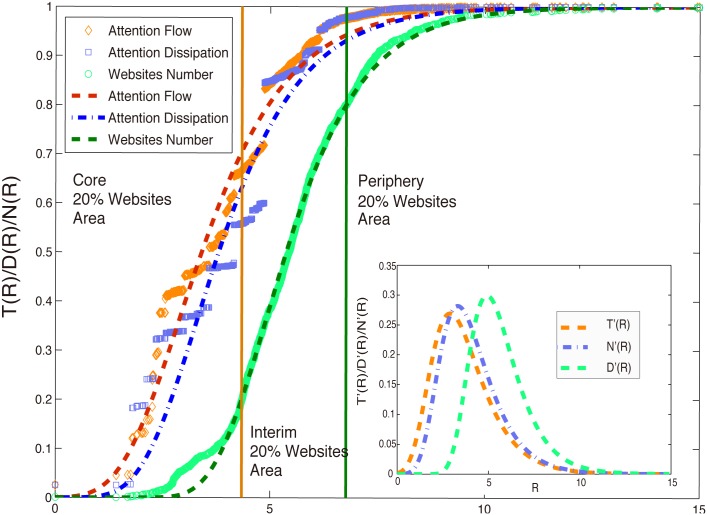
The normalized cumulative curves of attention flows (brown diamond), attention dissipations (blue squares), and the number of websites (green circles) along radius. The fitted normalized “S” curves for attention flows (dotted brown), dissipations (dashed and dotted blue), and the number of sites (dashed dark lines) are also shown. The inset shows the density curves of the three quantities and the derivatives to *R* of the three fitted “S” curves.

Because Google.com has the smallest average distance to other websites, it is set as the center of the geometric representation for all other websites which form a nearly symmetric ball around the center. Therefore, we study the distributions of the variables including attention flow (the traffic of each web site), attention dissipation (the flow to the sink from each web site), and the number of websites along the distance from the center of the ball. Instead of drawing the density curves of focal quantities directly, we accumulate them within the given radius to reduce the effect of noise in the data because cumulative curves are equivalent to density curves for distributions. We discover that with the increase of radius, the cumulative amounts of the quantities within the radius show sigmoid growth patterns (see Fig 5) and this S-curve pattern is very stable for different periods, namely October 10,2006, March 10,2007, September 10,2007 and February 10,2008. Details are shown in the supporting information.

These “S” curves reflect the heterogeneities of the distributions. From Fig 5, we know that most of quantities are concentrating in the central areas with radius 6. We then separate the whole ball into three layers along the radius according to the quantiles of the number of websites. The first layer is the ball with radius 4.1 which is selected according to the 20% quantile of the websites. That means 20% most important (popular) websites are included in this layer. However, it attracts almost three quarters of attention flows in the whole network with only a relatively small fraction of dissipations. Therefore we regard the layer as the core. In the second layer with radius in between 4.1 and 6.2 (the 80% quantile of websites), about 60% sites are included, but only 22% attention flows are contained with the cost of 38% dissipations. That means these websites are not attractive enough. We call this layer as the interim. Other small websites locate in the last layer, the periphery, which being of radii larger than 6.

To quantitatively characterize the “S”-shaped curves along radius for these three quantities, we use the gompertz function [40] to fit the normalized cumulative curves of attention flows (traffics, *T*(*R*)), attention dissipations (*D*(*R*)), and the number of websites (*N*(*R*)) within the radius *R*. The fitting functions can be expressed as:

$$\begin{array}{c} X\left(R\right)=\exp(-k_{X}\exp\left(-c_{X}R\right)), \end{array}$$ (9)

Where *X* can be *T*, *D*, *N*, and *k*_*X*_, *c*_*X*_, are the corresponding parameters to be estimated. And *c*_*X*_ characterizes the slope of the fast raising phase of the “S”-shaped curve, *k*_*X*_ indicates the offset of the whole curve along the *x* coordinate. The fitting results are shown in Table 4.

**Table 4:** The fitting results of “S” curves by using gompertze function.

	***k*_*X*_**	***c*_*X*_**	***R*^2^**
*T*(*R*) ∼ *R*	6.534 ± 0.167	0.7356 ± 0.043	0.9726
*D*(*R*) ∼ *R*	7.194 ± 0.337	0.6184 ± 0.010	0.9151
*D*(*R*) ∼ *T*(*R*)	75.650 ± 1.230	0.9146 ± 0.032	0.9977

### The relative growth of cumulative variables in the radial direction

**Fig 6:**
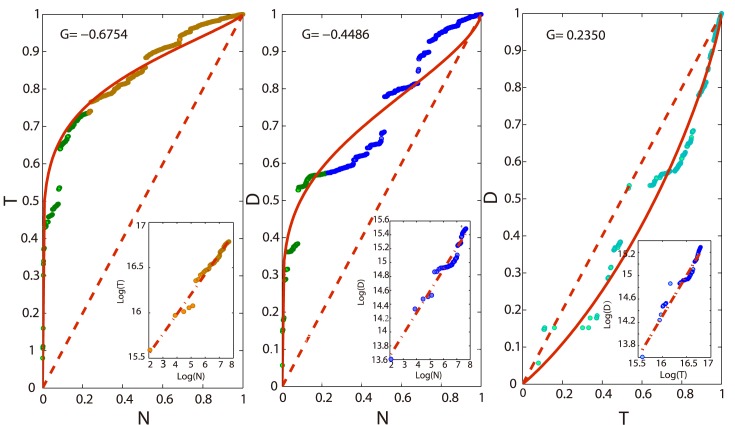
The Lorenze-liked curves and the GINI-liked coefficients among cumulative attention flows, attention dissipations, and the number of websites along the radius. The green nodes in the two sub-figures on the left mean the attention flows or dissipations of the 20% websites in the core. The insets show the log-log plots of the focal variable pairs.

To compare the relative rates of accumulation for different variables along radius, we can plot two variables together on one coordinate as shown in Fig 6. The curves can be also predicted theoretically by combining the gompertze functions together to eliminate *R*. For example, we consider the relationship between *N*(*R*) and *T*(*R*) and we know:

$$\begin{array}{c} \left\{ \begin{array}{c} N(R)=\exp(-k_{N}\exp\left(-c_{N}R\right))\\ T(R)=\exp(-k_{T}\exp\left(-c_{T}R\right)) \end{array}\right. \end{array}$$ (10)

After eliminating *R*, we have:

$$\begin{array}{c} T\left(R\right)=\exp(k_{T}{\left(-\frac{\ln(N(R))}{k_{N}}\right)}^{-\frac{c_{T}}{c_{N}}}) \end{array}$$ (11)

From Fig 6, we can read the relative speed of accumulation for any pair of variables. Thus, attention flow and dissipation accumulate faster than the number of websites along the radius. And the attention flow is faster than the dissipation. These curves resemble the Lorenze curve in income distribution which are named as Lorenze-liked curves in this paper. If the speeds of the two variables are the same, the curves collapse to the diagonals. And the bending degrees of curves reflect the differences on the speeds which can be quantified by a GINI-liked coefficient (*G*) defined as the difference (*A* − *B*) between the area (*A*) enclosed by the diagonal and the horizontal line and the area (*B*) enclosed by the fitting curve and the horizontal line. Therefore, if the fitting curve is above the diagonal, the GINI-liked coefficient is negative. The GINI-liked coefficients are shown in Fig 6.

Therefore, according to the Lorenze-liked curves, the amount of attention flow concentrates on the core layer, so it increases faster than the dissipation. The number of websites accumulates along the radius with a very slow speed compared to the other variables because the density peak appears in the second layer. Thus, very a few popular websites dominate the attention resources. And also, these websites are sticky enough so that the accumulative speed dissipation is slower than the attention flow.

### The Dynamics of the geometric representation

**Fig 7:**
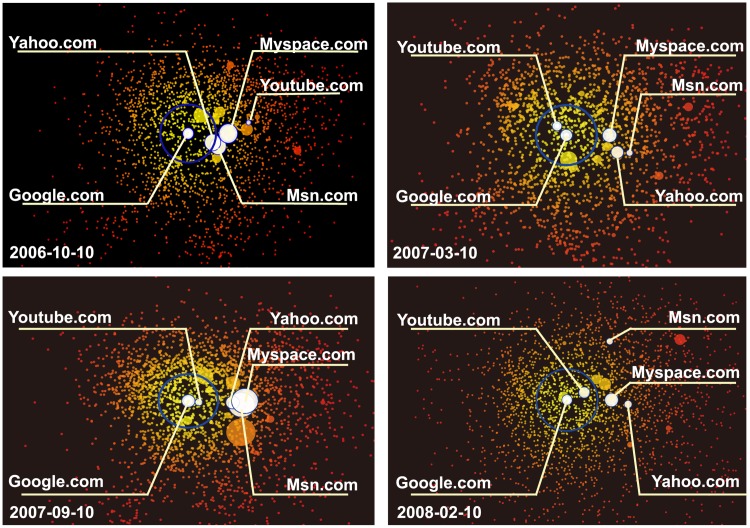
The snapshots of the geometric representations of the top 2200 websites in different days. The nodes of Google.com, Yahoo.com, Msn.com, Myspace.com and Youtube.com are highlighted. The radius of the blue circle is 1 which marks the central part of these websites.

Next, we study the dynamics of the representation. Four special snapshots for different times are selected such that the time spans between any two snapshots have similar lengths as shown in Fig 7.

It is interesting to observe that Google.com always locates in the center of the geometric representation in October 10,2006 and February 10,2008, while Yahoo.com and Msn.com go out of the central area of the map gradually. That indicates that Google.com has out-competed Yahoo.com and Msn.com to become the dominator of the search engine. However, after Youtube.com’s establishment on April 23, 2005, it quickly attracted large proportion of attention and run into the central area of the geometric representation, and become an important website in the entire attention ecosystem.

**Fig 8:**
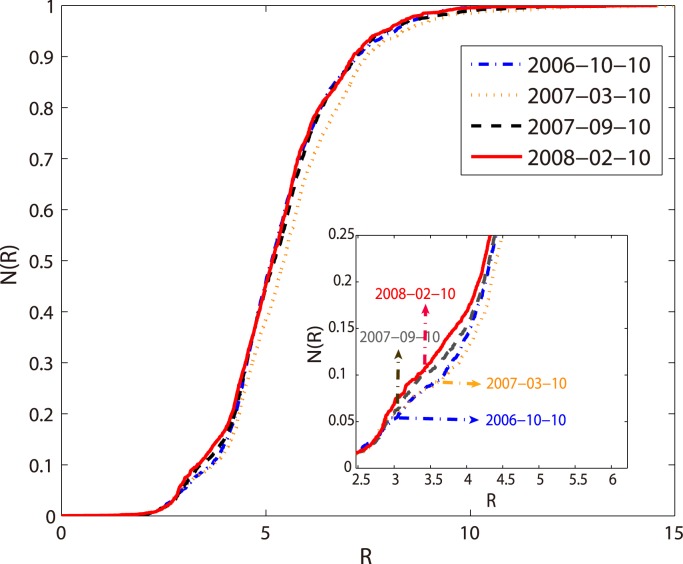
The “S” curves of the number of websites in different time by distinct colors.

Comparing the “S” curves between *N*(*R*) and *R* in different years, we can find that the “S” curves have slightly shifted over time for the part of *R* ≤ 5 as shown in Fig 8. This indicates that the central area of the system are becoming denser as time goes by indicating that the websites are closer and more connected each other. The distribution of attention flows, attention dissipations and websites within different time periods of a day are also discussed in the supporting information.

**Fig 9:**
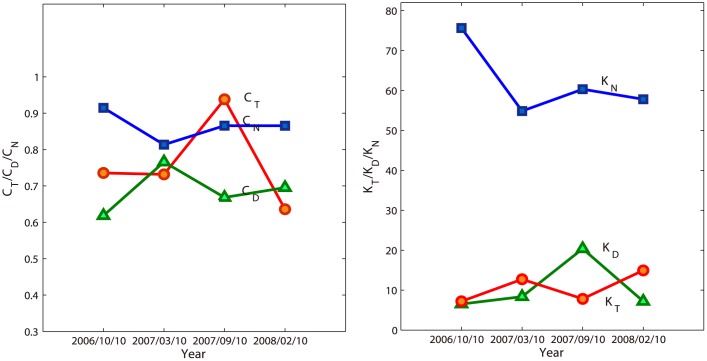
The variations of parameters, *c*_*T*_, *c*_*D*_, *c*_*N*_, *k*_*T*_, *k*_*F*_, *k*_*N*_ of “S” curve for selected snapshots.

The parameter *k* of gompertz function controls the translation of “S” curves and *c* controls the growth rate of the curves. From Fig 9, we can see that there is a downward trend from October 10, 2006 to February 10, 2008, corresponding to the cumulative curve of the number of websites offsets to left. It is also apparent that *c*_*N*_ is larger than *c*_*T*_, *c*_*D*_ in most situations, meaning that *N*(*R*) has more dramatic increases than *T*(*R*) and *D*(*R*) in general. This indicates the distribution of websites along the radius is always more heterogeneous.

**Fig 10:**
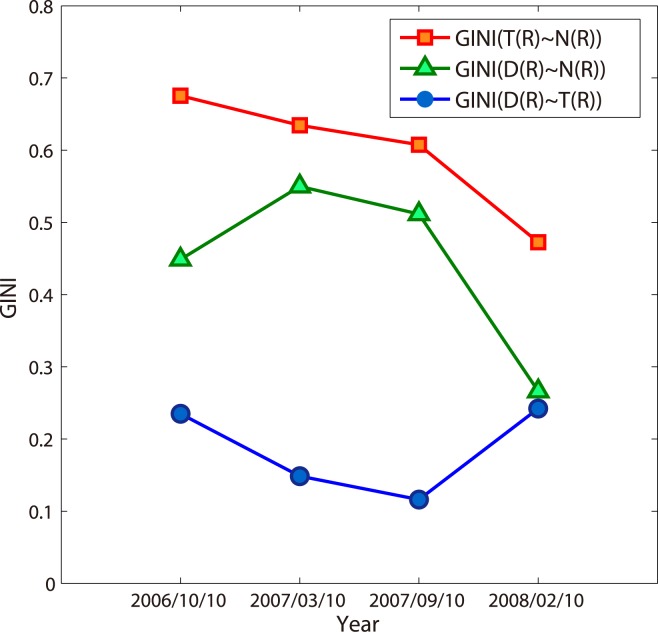
The GINI-liked coefficients’ variation during October 10,2006 and February 10,2008.

Furthermore, we consider the relative growth speeds of different cumulative variables along the radius in different snapshots which can be shown by the dynamics of the GINI-liked coefficients. From Fig 10, we read that the relative growth speeds in Fig 6 are almost kept. However the coefficient for *T* and *N* decreases continually which can be accounted by the left offset of the “S”-curve of the distribution for websites.

## Discussions and Conclusions

In this paper, we try to embed selected websites in a high dimensional space, and study the distributions of collective attention flows, dissipations on websites by using a biased collection of clickstream data. The geometric representation of the websites is based on a novel notion of flow distance defined on the underlying open flow network model of attention flow which integrates the topological structure of hyperlinks and the collective behavior of user traffics between sites. We find that although the social networks like Myspace.com and Facebook.com own most of the users’ attention, the most central website is the search engine—Google.com, in which the centrality is quantified by the average flow distances of the focal sites with all other websites.

We then focus on the collective distributions of websites, attention flows, and dissipations on the geometric representation space. We find that the geometric representation resembles an nearly symmetric ball, in which three different layers along the radius direction of the ball can be divided according to the distributions of attention flow and websites. The most inner layer, “core”, attracts 75% of attention flows and 55% dissipations by only 20% popular websites. While the second layer “interim” encloses most of normal websites (60%), but only 22% attention flows with 38% dissipations. The last layer, “periphery”, contains the left 20% websites with only 3% attention and 7% dissipations. Therefore, the distributions of attention flows, dissipations, and websites in the geometric representation are of great unevenness which can be well described by the “S”-shaped cumulative curve and the Lorenz-liked curve of relative growth along the distance from the center.

Finally, we show the general trends of dynamics of the representation by studying four snapshots of the geometric representations in different time points. We find that in general the distributions of attention flows, dissipations, and websites are stable along time. While a slight trend that websites trying to move inside the central area can be observed. And during the observable period, some websites like Msn.com and Yahoo.com gradually moves out of the central area, while Youtube.com moves toward the center.

Our research also have some drawbacks, for example, we only use the surfing records of one university to represent the traffics on the World Wide Web. The dataset is apparently biased and limited. However, we believe that our method and basic conclusions can be extended to larger data sets because this sample is representative. Second, the geometric representation needs to compute the pair-wised distances of all the websites, and the complexity increases in an *N* ^2^ speed. Further, the matrix inversion operation is needed when we compute the flow distance. This will make the whole task very tough when *N* is large. Therefore, some approximate methods such as Monte Carlo simulation are deserved.

Our work has some potential applications. First, the methodology can be applied to other fields rather than the clickstream data. The geometric representation can at least provide a good visualization for open flow networks. Second, our work may give an alternative evaluation for websites which is apparently distinguished with PageRank method and the traffic data. This evaluation can reflect both the link structure of websites and the collective behaviors of users. Third, the flow distance between two websites can also provide information of indirect interactions. This may outperform the traditional analysis approaches which merely focusing on directed upcoming websites, and help web masters to post their advertisement in more appropriate places.

## Supporting Information

S1 File Supporting files. Fig A, The example to illustrate the basic idea of embedding algorithm. Fig B, The positions’ variation over the computation iterations. Fig C, The normalized cumulative curves of attention flows (brown diamond), attention dissipations (blue squares) and the number of websites (green circles) along radius on March 10, 2007. The inset shows the density curves of the three quantities and the derivatives to *R* of the three fitted “S” curves. Fig D, The Lorenze-liked curves and the GINI-liked coefficients among cumulative attention flows, attention dissipations, and the number of websites along the radius on March 10, 2007. The green nodes in the two sub-figures on the left represent the attention flows or dissipations of the 20% websites in the core. The insets show the log-log plots of the focal variable pairs. Fig E, The normalized cumulative curves of attention flows (brown diamond), attention dissipations (blue squares) and the number of websites (green circles) along radius on September 10, 2007. The inset shows the density curves of the three quantities and the derivatives to *R* of the three fitted “S” curves. Fig F, The Lorenze-liked curves and the GINI-liked coefficients among cumulative attention flows, attention dissipations, and the number of websites along the radius on September 10, 2007. The green nodes in the two sub-figures on the left represent the attention flows or dissipations of the 20% websites in the core. The insets show the log-log plots of the focal variable pairs. Fig G, The normalized cumulative curves of attention flows (brown diamond), attention dissipations (blue squares) and the number of websites (green circles) along radius on February 10, 2008. The inset shows the density curves of the three quantities and the derivatives to *R* of the three fitted “S” curves. Fig H, The Lorenze-liked curves and the GINI-liked coefficients among cumulative attention flows, attention dissipations, and the number of websites along the radius on February 10, 2008. The green nodes in the two sub-figures on the left represent the attention flows or dissipations of the 20% websites in the core. The insets show the log-log plots of the focal variable pairs. Fig I, The special case in calculating GINI-liked coefficients. Fig J, The distribution of attention flows on October 10, 2006. Fig K, The distribution of attention dissipations on October 10, 2006. Fig L, The distribution of websites on October 10, 2006.
(PDF)
