# Supplementary material for: A Geometric Representation of Collective Attention Flows
Source: PLoS One. 2015 Sep 1;10(9):e0136243. doi: 10.1371/journal.pone.0136243 (PMC4556699; doi:10.1371/journal.pone.0136243)
Supplement: S1 File — Fig A, The example to illustrate the basic idea of embedding algorithm. Fig B, The positions’ variation over the computation iterations. Fig C, The normalized cumulative curves of attention flows (brown diamond), attention dissipations (blue squares) and the number of websites (green circles) along radius on March 10, 2007. The inset shows the density curves of the three quantities and the derivatives to R of the three fitted “S” curves. Fig D, The Lorenze-liked curves and the GINI-liked coefficients among cumulative attention flows, attention dissipations, and the number of websites along the radius on March 10, 2007. The green nodes in the two sub-figures on the left represent the attention flows or dissipations of the 20% websites in the core. The insets show the log-log plots of the focal variable pairs. Fig E, The normalized cumulative curves of attention flows (brown diamond), attention dissipations (blue squares) and the number of websites (green circles) along radius on September 10, 2007. The inset shows the density curves of the three quantities and the derivatives to R of the three fitted “S” curves. Fig F, The Lorenze-liked curves and the GINI-liked coefficients among cumulative attention flows, attention dissipations, and the number of websites along the radius on September 10, 2007. The green nodes in the two sub-figures on the left represent the attention flows or dissipations of the 20% websites in the core. The insets show the log-log plots of the focal variable pairs. Fig G, The normalized cumulative curves of attention flows (brown diamond), attention dissipations (blue squares) and the number of websites (green circles) along radius on February 10, 2008. The inset shows the density curves of the three quantities and the derivatives to R of the three fitted “S” curves. Fig H, The Lorenze-liked curves and the GINI-liked coefficients among cumulative attention flows, attention dissipations, and the number of websites along the radius on February 10, [file pone.0136243.s001.pdf]

Supporting Information of "A Geometric Representation of Collective Attention Flows"

Peiteng Shi, Xiaohan Huang, Jun Wang, Jiang Zhang, Su Deng, Yahui Wu

1. The implementation of flow distance L_{ij}

Distance on graph is a very useful concept [1]. Both the shortest path distance [2], resistance distance [3] and the mean first-passage distance of a random walker [4-7] can reflect the intrinsic properties of the graph.

However, conventional first-passage distance on a graph is based on the basic assumption that the whole network is closed, which means the random walker cannot escape from the network, thus the total number of walkers on the graph is conservative. Nevertheless, the open flow network is an open system. Random walkers can flow into the system from the source and flow out to the sink despite the total number of walkers staying in the network can be also conservative if the flow system is in a steady state. Therefore, the traditional method for closed system cannot be simply extended to open flow networks. It is necessary to extend the distance notions for open flow networks.

1.1 The conventional first-passage random walk distance

In closed network, the conventional first-passage random walk distance from i to j on a Markov chain is calculated as follows [8]. First, we delete the corresponding row and column of node j from the original Markov chain M to derive a new matrix M_{-j}. And we can calculate the random walk distances from any node to j according to the equation

$$H(.,j) = (I - M_{-j})^{-1} e \tag{1}$$

Where $H(.,j)$ is a vector representing the random walk distances from any node to j, and $e = (1,\ 1, \ldots,\ 1)^T$. Therefore the distance from i to j is $H(i,j)$, the ith element in $H(.,j)$. Actually, when the corresponding row and column of node j are deleted, the original closed flow network is converted to an open flow network by treating node j as a sink. However, this method cannot be extended to an open flow network directly because there already exists one sink node $N+1$ already. And when the corresponding column and row of node j are deleted, the Markov chain may have two absorbing states (sinks), therefore, Eq.(1) is not correct. We must use other method to calculate the flow distances on open flow networks.

1.2 The first-passage flow distance

We define the total flow from i to j denoted as ρ_{ij} is defined as the total number of particles that have been visited i and arrive at j in each time no matter if it is the first time or not. And the average step that these particles have jumped is defined as the total flow distance which is denoted by t_{ij}

Then, the first-passage flow from i to j can be denoted by ϕ_{ij} as the number of particles that reach j in each time step for the first time and have been visited i. The average step that these particles have jumped is defined as the first-passage flow distance, denoted by l_{ij}.

Actually, we can deduce the explicit expression of various flow distances conveniently once the total flow and first-passage flow expressions are given [9-11].

First, according to the definition of the total flow from i to j along all possible paths, we have

$$t_{ij} = \sum_{k=1}^{\infty} k p_{ij}^{k} \tag{2}$$

Where p_{ij}^k denotes the probability that particles transfer from i to j after k steps. One may think $p_{ij}^k = (M^k)_{ij}$, however, it is not the true because p_{ij}^k is normalized for all paths with all possible lengths k, i.e., $\sum_{k=1}^{\infty} p_{ij}^k = 1$. However, $(M^k)_{ij}$ is normalized for all js, i.e., $\sum_{j=0}^{N+1} (M^k)_{ij} = 1$. We know that the flow from i to j after k steps is $\phi_{0i}(M^k)_{ij}$ and the total flow along all possible paths is ρ_{ij}, therefore,

$$p_{ij}^k = \frac{\phi_{0i}(M^k)_{ij}}{\rho_{ij}} \tag{3}$$

Thus bringing this equation to Eq.(2), we have,

$$\begin{aligned} t_{ij} &= \sum_{k=1}^{\infty} k \frac{\phi_{0i}(M^k)_{ij}}{\rho_{ij}} = \frac{\phi_{0i}(\sum_{k=1}^{\infty} k M^k)_{ij}}{\rho_{ij}} \\ &= \frac{\phi_{0i}(MU^2)_{ij}}{\rho_{ij}} = \frac{\phi_{0i}(MU^2)_{ij}}{\phi_{0i} u_{ij}} \\ &= \frac{(MU^2)_{ij}}{u_{ij}} \end{aligned} \tag{4}$$

In which, we have used the following series expansion:

$$MU^2 = M\left(\frac{1}{I-M}\right)^2 = \sum_{k=1}^{\infty} k M^k \tag{5}$$

The distance t_{ij} exists only if there is a connected path from i to j. If the path exists, then $u_{ij} > 0$.

Similarly, we can obtain the expression for first passage flow distance. First, according to the definition of the first-passage distance from i to j, we have

$$l_{ij} = \sum_{k=1}^{\infty} k q_{ij}^{k} \tag{6}$$

Where q_{ij}^{k} denotes the probability that particles jump from i to j after k steps in the first time. One cannot use p_{ij} (Eq. (3)) because it contains the circulation flow from j to i. Let us assume that all the particles arriving at j will be removed from the system, that is to say, we assume that j is another sink, then all the calculations for the total flow distance is correct. To make this point clear, we define a new matrix M_{-j} as:

$$(M_{-j})_{rs} = \begin{cases} m_{rs}, & r \neq j \\ 0, & r = j. \end{cases} \tag{7}$$

And the correct expression for the probability q_{ij}^{k} is

$$q_{ij}^{k} = \frac{\phi_{0i}(M_{-j}^{k})_{ij}}{\phi_{ij}} \tag{8}$$

Insert it into Eq. (6), we have

$$l_{ij} = \frac{u_{jj}(M_{-j}U_{-j}^{2})_{ij}}{u_{ij}} \tag{9}$$

According to the theory in [12], when $u_{ij} \neq 0$ (i connects to j), this formula can be reduced to

$$l_{ij} = \frac{(MU^{2})_{ij}}{u_{ij}} - \frac{(MU^{2})_{jj}}{u_{jj}} = t_{ij} - t_{jj} \tag{10}$$

Therefore, the difference between t_{ij} and l_{ij} is just the total flow distance from j to j. The matrix $(t_{jj})_{(N+1)\times(N+1)}$ have identical rows. It quantities the ability of self-circulation of each node in the system.

The flow distances between any given two websites i and j reflect the average number of transition steps of a random walker along the network from i to j for the first time. Hence, the closer the websites, the more easily the users jump to another website from one website, the

more possible they contain relevant information.

2. The Detailed Interpretation of Embedding Methods

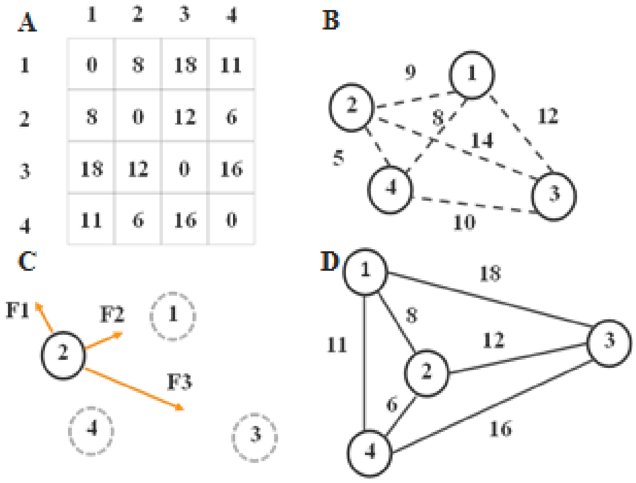

Figure A. The example to illustrate the basic idea of embedding algorithm.

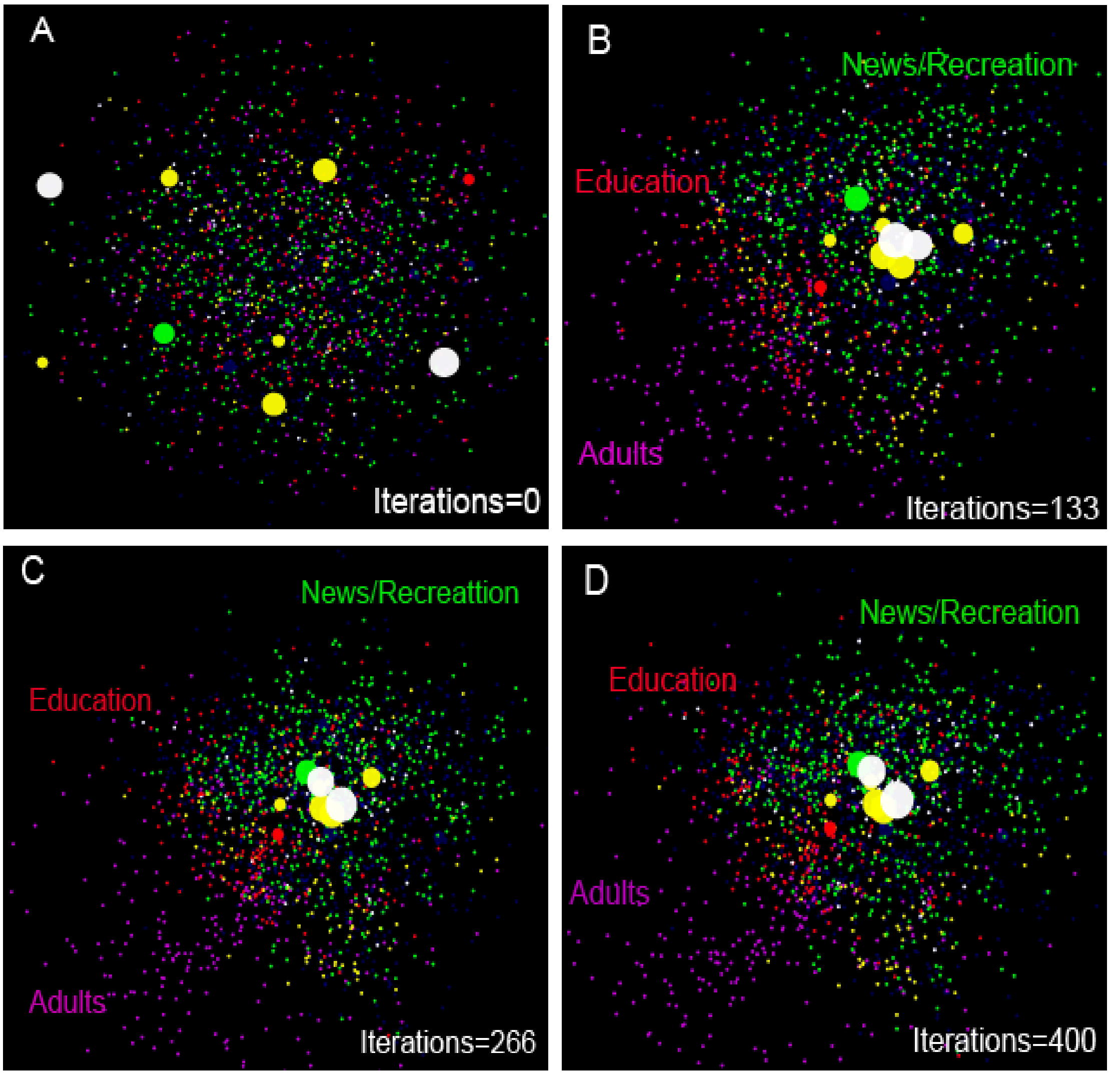

Figure B. The positions' variation over the computation iterations.

Suppose there are four websites and the flow distances among them are shown in Fig. A(A). To embed each website into the Euclidean space and assign one "geometry imagine" for every website, we first allocate the nodes randomly in the Euclidean space, taking 2-dimensional space as an example. Fig. A(B) means the random positions of the four websites and the number beside the dashed line is the Euclidean distance of two websites.

Then, we can use the BigBang method [13] to adjust the nodes positions, making difference between the Euclidean distance and the flow distances among websites as small as possible. For example, the Euclidean distance between website 1 and website 2 is larger than the flow distance which is 8. According to the spring algorithm [14], the natural length of the spring is 8 and the spring between website 1 and website 2 is stretched. Then, website 2 will get a pull (F2) from website 1 to deduce the Euclidean distance. In a similar way, the Euclidean distance between website 2 and website 3 is larger than the flow distance, website 2 will get a pull from website 3, too. While the distance between website 2 and website 1 is less than the flow distance, so the website 2 will receive a repulsion to make the Euclidean distance between them larger.

Fig. A(C) has marked the force exerted on website 2 and website 2 will move on the direction of resultant force. The distance moving on is proportional to the strength of resultant force.

Fig. B shows the positions variation of top 2200 websites on October 10 in 2006 with the computation iterations. The sub-figures are the websites positions in 2-dimensional space at different number of iterations. It's obvious to see that the websites are randomly positioned in the figure firstly. Then, every node will change its position according to the resultant force it gets to approach its near neighbors in flow distances matrix. When the number of iterations equals 133, the websites with large amount of traffic go to the central part of the system and three regions appear: Education, Adults and News/Recreation. With the increase of iterations number, the websites belonging to the same category are closer to each other. This indicates that the websites close to each other are more likely to contain similar information.

3. Universal pattern in the geometry of WWW

The S curves of attention flow, attention dissipation and websites with the radius of WWW and the relative growth of cumulative variables in the radial direction at different times are shown blow, together with the relationship among the three variables. For the data loss on June 10, 2006, we select four days evenly during October 10, 2006 and February 10, 2008 to analyze.

3.1 The universal pattern on March 10, 2007, September 10, 2007 and February 10, 2008

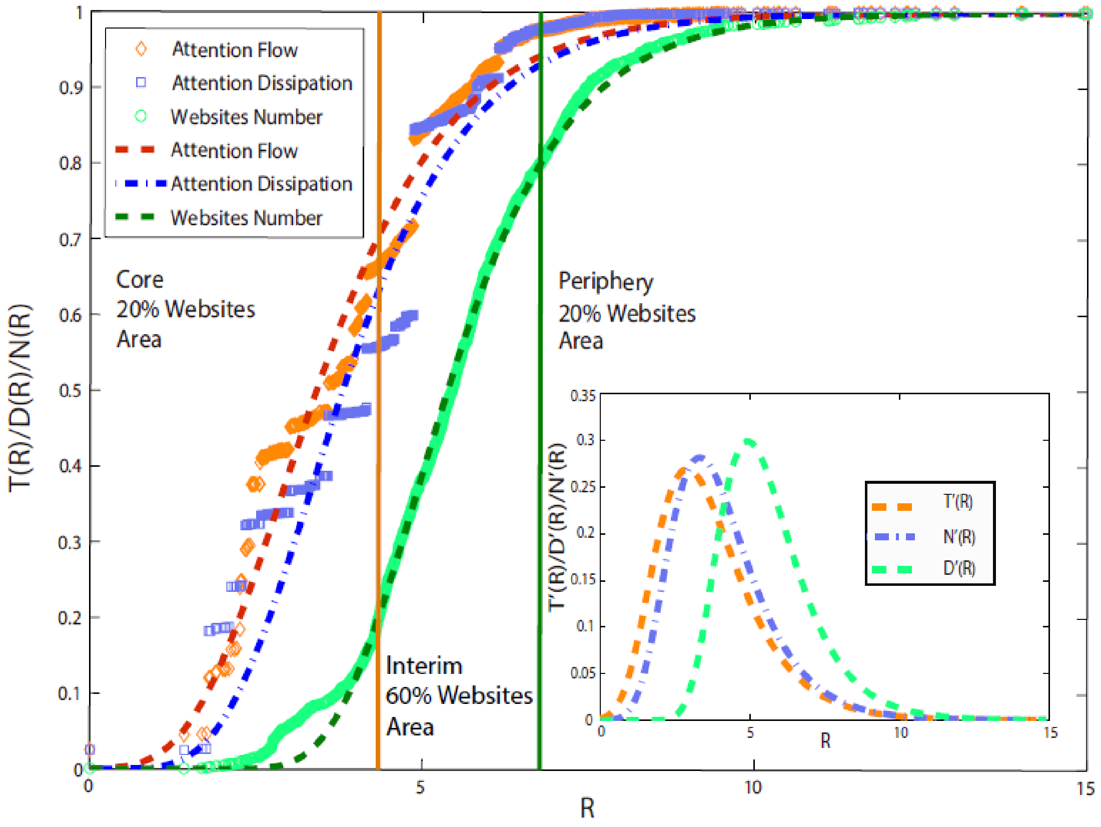

Figure C. The normalized cumulative curves of attention flows (brown diamond), attention dissipations (blue squares) and the number of websites (green circles) along radius on March 10, 2007. The inset shows the density curves of the three quantities and the derivatives to R of the three fitted "S" curves.

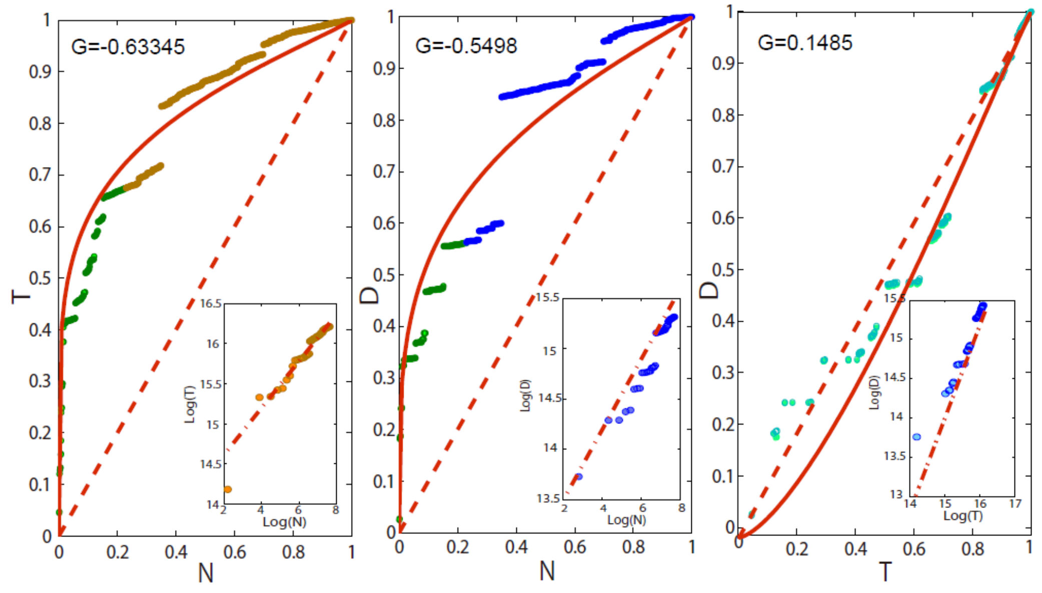

Figure D. The Lorenze-liked curves and the GINI-liked coefficients among cumulative attention flows, attention dissipations, and the number of websites along the radius on March 10, 2007. The green nodes in the two sub-figures on the left represent the attention flows or dissipations of the 20% websites in the core. The insets show the log-log plots of the focal variable pairs.

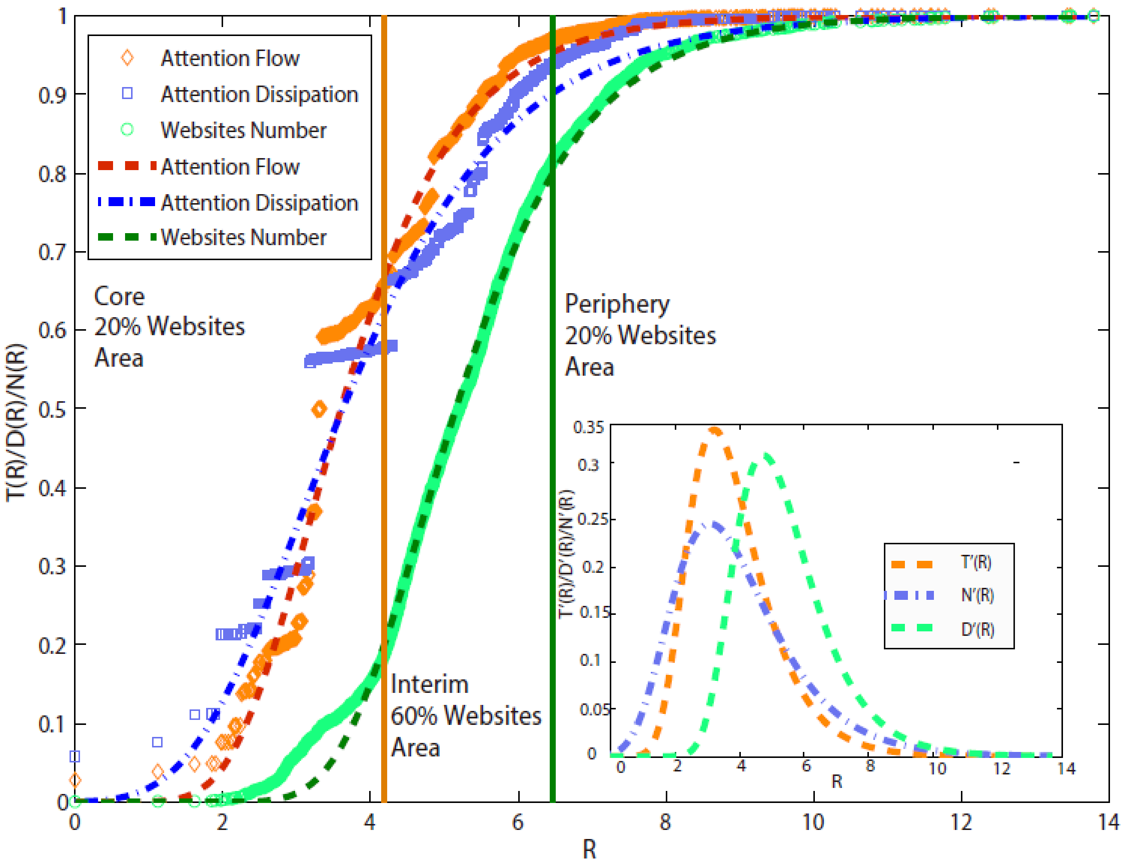

Figure E. The normalized cumulative curves of attention flows (brown diamond), attention dissipations (blue squares) and the number of websites (green circles) along radius on September 10, 2007. The inset shows the density curves of the three quantities and the derivatives to R of the three fitted "S" curves.

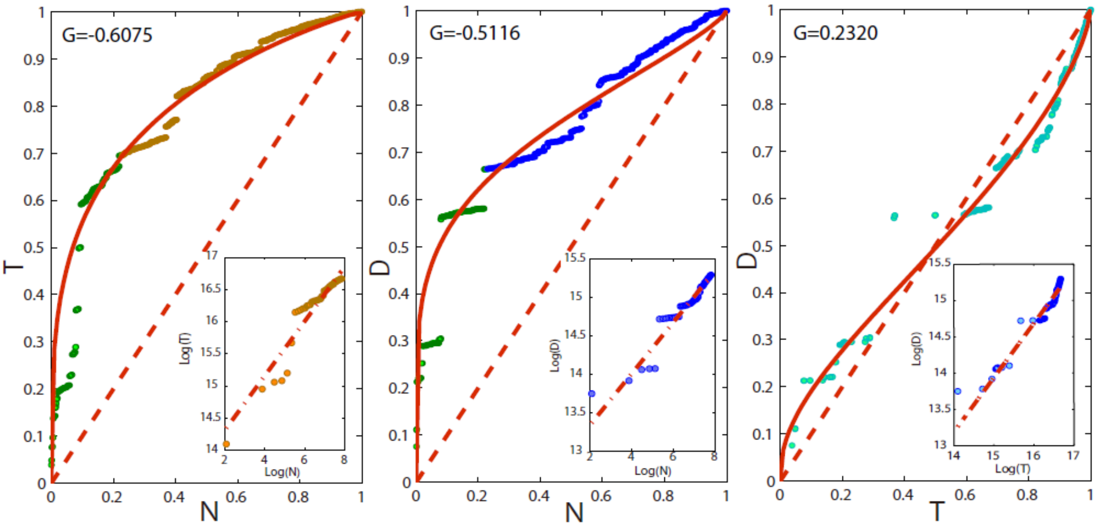

s

Figure F: The Lorenze-liked curves and the GINI-liked coefficients among cumulative attention flows, attention dissipations, and the number of websites along the radius on September 10, 20007. The green nodes in the two sub-figures on the left represent the attention flows or dissipations of the 20% websites in the core. The insets show the log-log plots of the focal variable pairs.

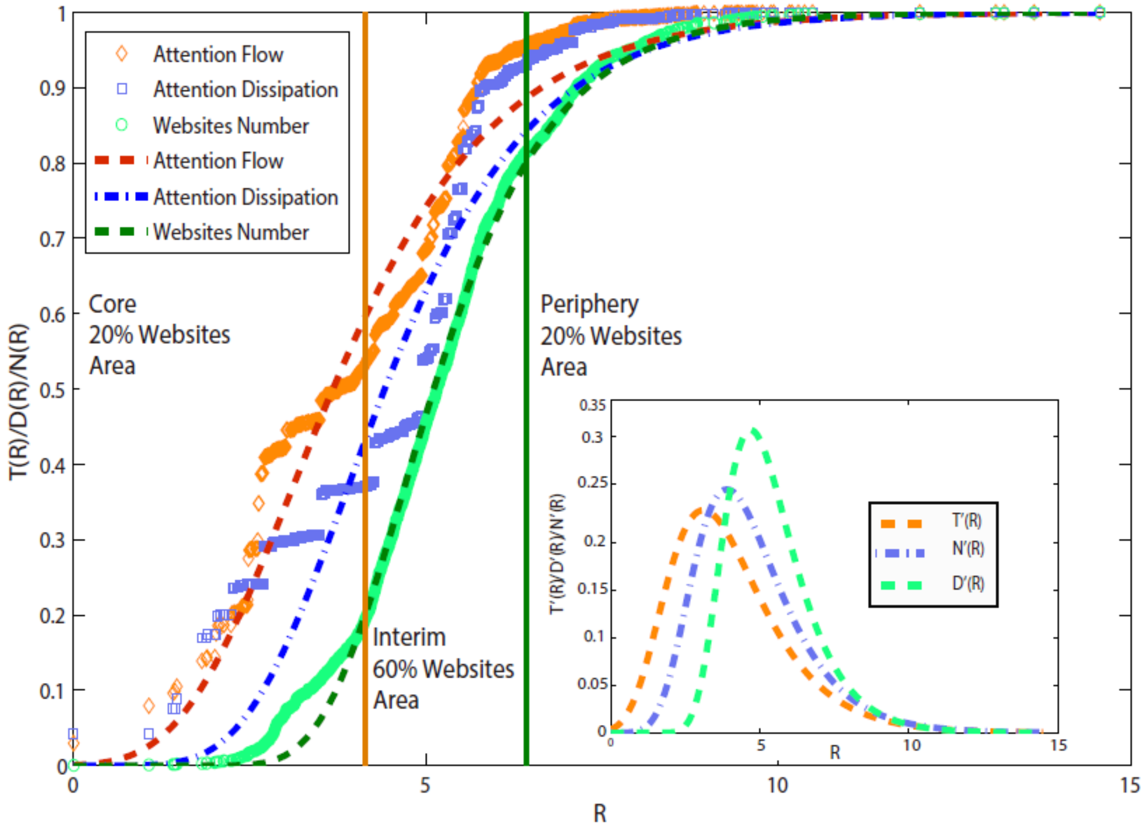

Figure G. The normalized cumulative curves of attention flows (brown diamond), attention dissipations (blue squares) and the number of websites (green circles) along radius on February 10, 2008. The inset shows the density curves of the three quantities and the derivatives to R of the three fitted "S" curves.

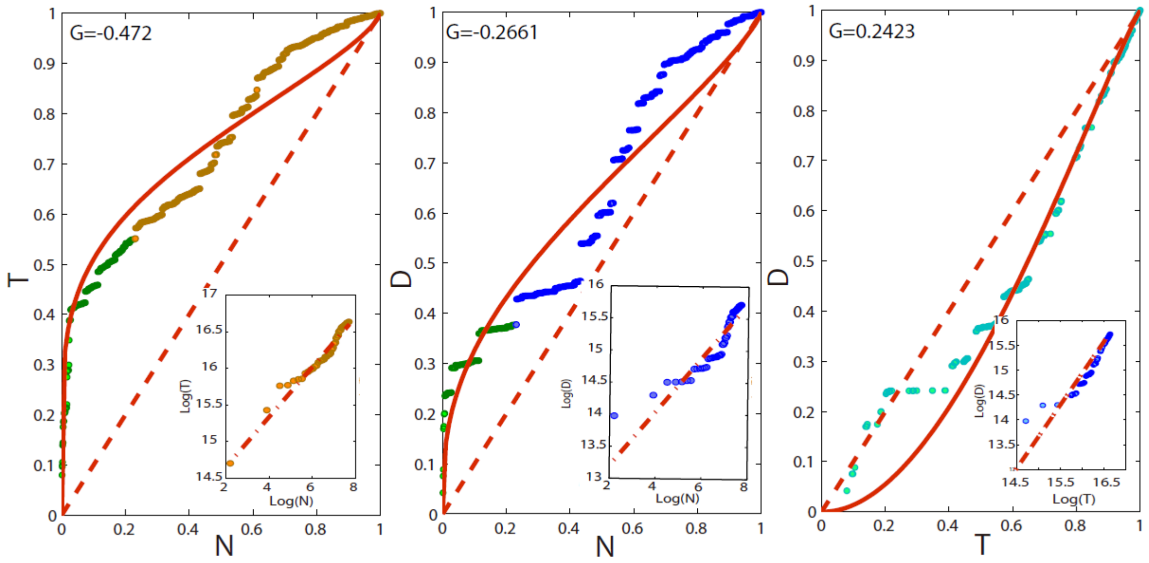

Figure H. The Lorenze-liked curves and the GINI-liked coefficients among cumulative attention flows, attention dissipations, and the number of websites along the radius on September 10, 2007. The green nodes in the two sub-figures on the left represent the attention flows or dissipations of the 20% websites in the core. The insets show the log-log plots of the focal variable pairs.

From Fig. C, Fig. E and Fig. G, it's obviously that attention flows, attention dissipations and websites all follow the growth of S curve with the increase of R in the geometric representation and it's a universal pattern in the distributions of attention flows, attention dissipations and websites.

All the distributions of websites, attention flows, and dissipations can be divided into three spherical crowns (core, interim, and periphery). 20% popular sites (Google.com, Myspace.com, Facebook.com, etc.) attracting 68.75% ((75%+70%+65%+55%)/4=66.3%) attention flows with only 51.5% ((55%+55%+58%+38%)/4=51.5%) dissipations (log off users) locate in the central layer with the radius about 4.1 in average. While 60% sites attracting only about 31.5% ((22%+28%+33%+43%)/4=31.5%) traffics with almost 38.4% ((38%+38%+37%+40.7%))/4= 38.4%) dissipations locate in the middle area with radius in between 4.1 and 6.3. Other 20\% sites only own 2% ((2%+2%+2%+2%)/4=2%) attention flows and 3.5% ((2%+2%+5%+5%)/4=3.5%) attention dissipations in average. A small proportion of websites in the core part attract a large proportion of attention flows.

What's more, there are much more proportion of attention flows than attention dissipations in the core part. We can also find that the most intensive position of attention flows is closer to the center than the most intensive position of attention dissipations, which can be read from the insets of Fig. C, Fig. E and Fig. G that the maximum of $T'(R)$ comes a little earlier than the maximum of $D'(R)$ with the increase of R. This indicates that the "attraction" and "stickness" of WWW.

Fig. D, Fig. F and Fig. H presents the Lorenze-liked curves and the GINI-liked coefficients among cumulative attention flows, attention dissipations, and the number of websites along the radius at different times. The green nodes in the left two sub-figures in Fig. D, Fig. F and Fig. H mean the proportion of attention flows or dissipations. The accumulative attention flows and attention dissipations of 20% websites in the core grow much faster than other 80% websites. Most of the attention flows and dissipations are concentrated in the core part. What's more, the GINI-liked coefficients of the relationship between attention flows and websites are always larger than the relationship between attention dissipations and websites, revealing the attention flows distributed more unevenly than the attention dissipations.

3.2 The calculation of GINI-liked coefficients in one special situation

The GINI-liked coefficient G is defined as 2(A-B), which A is the area enclosed by the diagonal and the horizontal line and B is the area enclosed by the fitting curve and the horizontal line.

It's deserve to notice that the line shown on Fig. I is one special case in calculating the GINI-liked coefficient and this situation happens on the third sub-figure of Fig. F. There are two parts of the fitted line on Fig. I.

The fitted line on the left part is above the diagonal line and the fitted line on the right part is below the diagonal line. Hence, the GINI-liked coefficient of the left part (2(A-B)) is positive while coefficient of right part (2(A'-B')) is negative. If we neglect this point, the positive part and the negative part will offset and the GINI-liked coefficient value of the whole curve will not be proportional to the area of (-(A-B)+(A'-B')) but the area of (-(A-B)-(A'-B')).s

The GINI-liked coefficient represents the degree of irregularity of one variable's distribution.

The overall irregularity should consider the irregularity of two sub-parts together. Therefore, we calculate the GINI-liked coefficient by adding up the absolute GINI-liked coefficient values of two parts.

Thus, the GINI-liked coefficient for this kind of curve is 2 (-(A-B)+(A'-B').

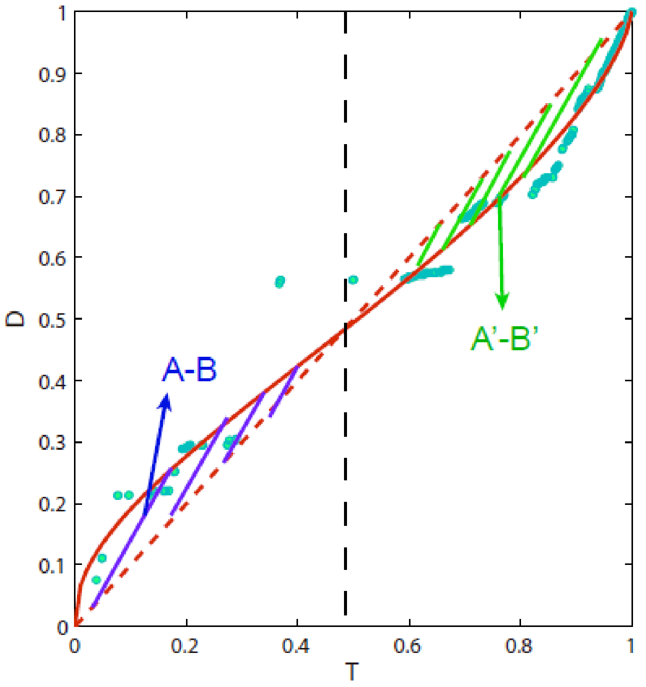

Figure I. The special case in calculating GINI-liked coefficients.

4. The distribution of attention flows, attention dissipations and websites within different time periods of a day

We divide one day into 4 parts to see that wether attention flows, dissipations & websites are distributed differently in different time buckets. 0:00~6:00 is regarded as the Midnight Time, 6:00~12:00 is regarded as the Morning Time, 12:00~18:00 is taken as the Afternoon Time, and the rest, 18:00~0:00, is the Evening Time.

After calculating the flow distances among websites and getting the geometry represeatations of three variables during the four periods, we can obtain the distribution of them.

In order to show the main different features, we investigate the distribution of one variable using two methods. One is $X(R)(Expressed\ as\ percent)$, which and another is $X(R)(Expressed\ as\ quantity)$, where X can be replaced by $T(attention\ \ flow)$, $D(attention\ dissipation)$ & $N(websites\ number)$. Taking attention flow as an example, $T(R)(Expressed\ as\ percent)$ means the percentage of total attention flows that the websites within radius R occupy, while $X(R)(Expressed\ as\ quantity)$ represents the amount of attenton flows within radius R.

Hence, for the two indicators, the fomer reveals the accumulating speed of X, while the latter depicts the accumulating quantity.

4.1 The distribution of attention flows during four periods

From Fig. J, we can see that the distibution of attention flows on attention flows are almost the same, indicating that the accumulating speeds of them are the same with each other. While the subfigure on the right shows the distinct difference of attention flows on Midnight Time, Morning Time, Afternoon Time and Evening Time. The attention flows of the Midnight time from 0:00 to 6:00 is smallest. A few people are active on the internet. At the same time, attentio flows on Morning Time from 6:00 to 12:00 are the second-smallest. People often begin to work from 8:00 and people are going into the internet world gradually. It’s easy to see that attention flows on the Afternoon Time fron 12:00 to 18:00 ranks top-one, and people are most active during this time. Evening Time from 18：00 to 0:00 owns the second-largest attention flows.

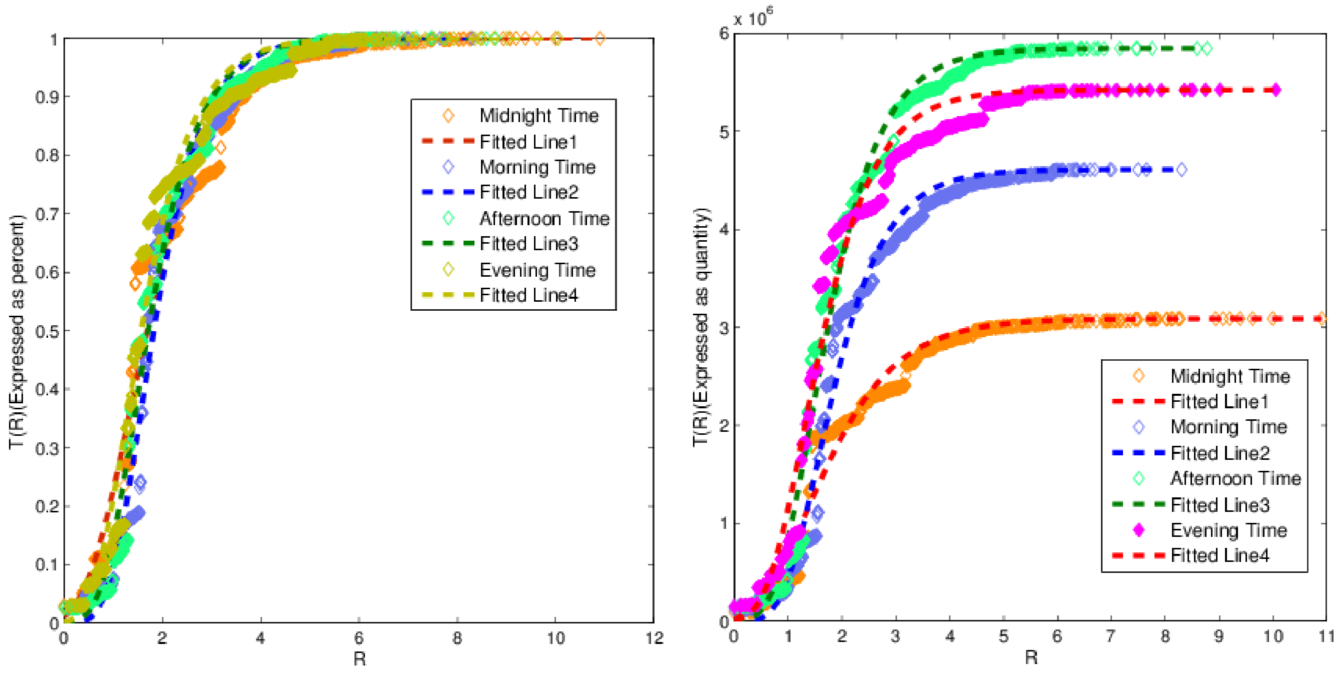

Figure J. The distribution of attention flows on October 10, 2006.

4.2 The distribution of attention dissipations during four periods

The distribution of attention dissipations are also analysied. Obviously, the distribution line of Midnight Time & Morning Time are above that of Afternoon Time & Evening Time, depicting that the dissipation speed during the Evening Time & Midnight Time are quickier than that of during the other two periods. It’s also deserve to notice that the Evening Time doesn’t own most attention flows but most attention dissipations.

Usually, people won’t click the websites continously when they are tired in the night, which may results in the greater dissipation speed during the Evening & Midnight Time from 18:00 to 6:00 tomorrow. More percentage of contimous access to websites behaviors happen during the Morning Time & Afternoon Time, which are the work time of people. It might show that people usually search information on the internet by visiting websites one by one in their work time, while in their Evening & Midnight Time, they usually choose to relax themselves by just enjoying one specific website like one movie website or some websites else.

Of course, much more attention flows are in the Afternoon Time. So the total dissipations are still larger than that of the Morning Time and Midnight Time.

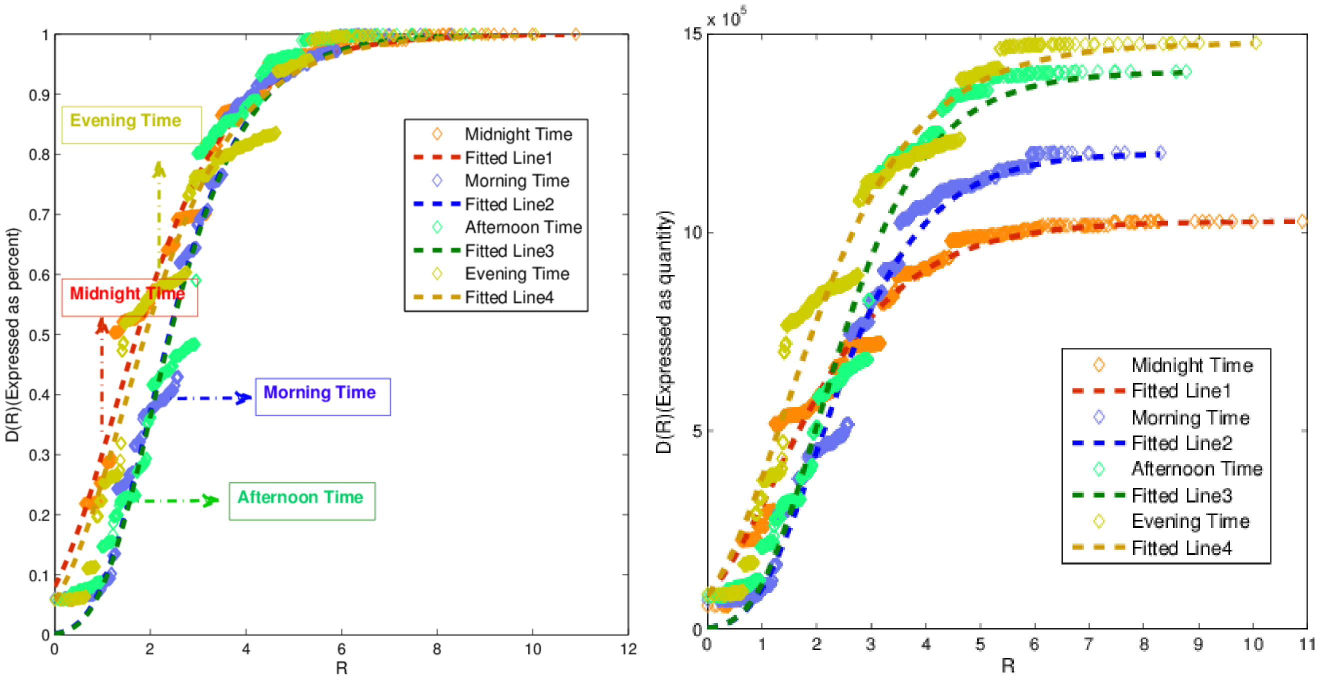

Figure K. The distribution of attention dissipations on October 10, 2006.

4.3 The distribution of websites during four periods

It's similar with the situation of attention flows that the distribution lines of websites during four time buckets almost concide each other, revealing the same accuating speed of websites. Due to less surfing behaviors in the Midnight Time, the websites at that time is also the least. On the contrary, people are most active in the Afternon Time, more websites are visited.

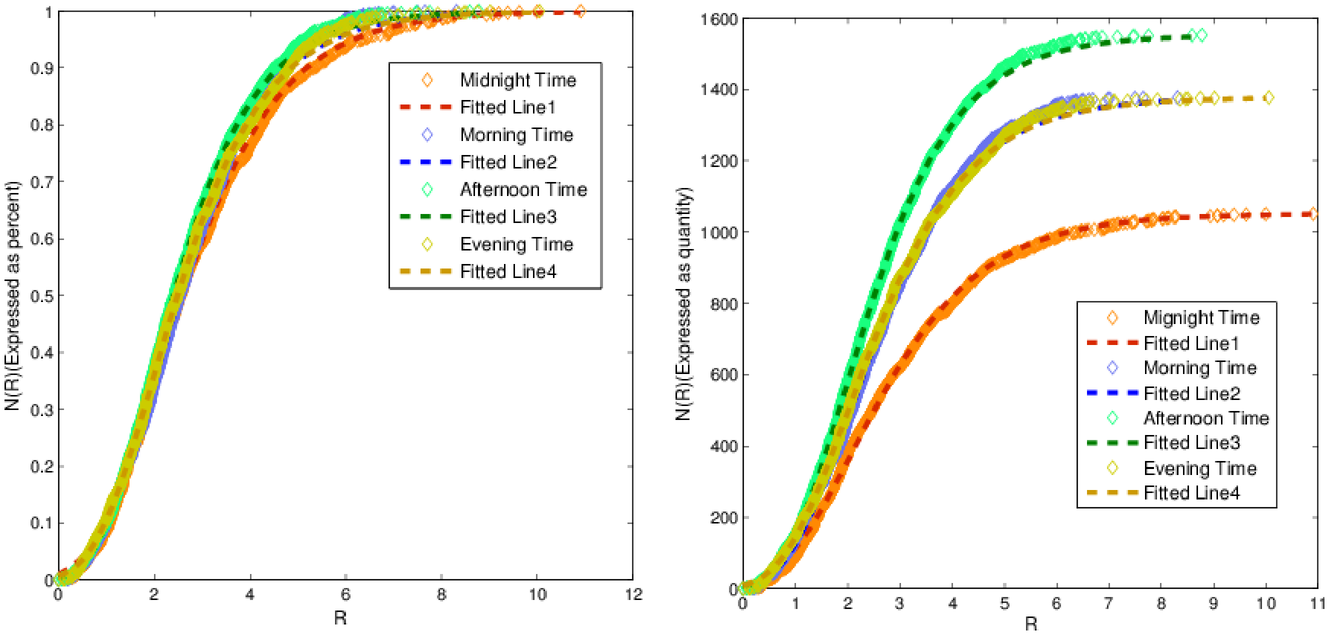

Figure L. The distribution of websites on October 10, 2006.

In a word, by investigating the distribution of attention flows, attention dissipations and websites, we can obtain that people are most active in the Afternoon Time, follwed by Evening Time, Morning Time and Midnight Time. Ads may get more attention in the Afternoon Time. Besides, attention dissipations are larger in the Evening Time than the other three time buckets.

Reference:

[1]D. Brockmann, D. Helbing. The hidden geometry of complex, network-driven contagion phenomena[J]. Science, 2013, 342(6164): 1337-1342.

[2]B. V. Cherkassky, A. V. Goldberg, T. Radzik, Shortest paths algorithms: Theory and experimental evaluation[J]. Mathematical programming, 1996, 73(2): 129-174.

[3]D. J. Klein, M. Randić. Resistance distance[J]. Journal of Mathematical Chemistry, 1993, 12(1): 81-95.

[4] J. D. Noh, H. Rieger, Random walks on complex networks, Physical review letters, 2004, 92(11): 118701.

[5] F. Blöchl, F. J. Theis, F. Vega-Redondo, E. O. Fisher, Vertex centralities in input-output networks reveal the structure of modern economies[J]. Physical Review E, 2011, 83(4): 046127.

[6] L. Lovász, Random walks on graphs: A survey [J]. Combinatorics, Paul erdos is eighty, 1993, 2(1): 1-46.

[7] P. Tetali, Random walks and the effective resistance of networks[J]. Journal of Theoretical Probability, 1991, 4(1): 101-109.

[8] B. D. Hughes. Random walks and random environments[J]. 1996.

[9] R. E. Ulanowicz, Quantitative methods for ecological network analysis[J]. Computational Biology and Chemistry, 2004, 28(5): 321-339.

[10] J. Zhang, L. Guo, Scaling behaviors of weighted food webs as energy transportation networks[J]. Journal of theoretical biology, 2010, 264(3): 760-770.

[11] J. Zhang, L. Wu, Allometry and dissipation of ecological flow networks[J]. PloS one, 2013, 8(9): e72525.

[12] B. Shen, J. Zhang, Q. Zheng. Exploring multi-layer flow network of international trade based on flow distances[J]. arXiv preprint arXiv:1504.02361, 2015.

[13] Y. Shavitt, T. Tankel, Big-bang simulation for embedding network distances in euclidean space[J]. IEEE/ACM Transactions on Networking (TON), 2004, 12(6): 993-1006.

[14] W.T. Tutte, How to draw a graph[J]. Proc. London Math.Soc, 1963, 13(3): 743-768.
